# Phenotype Switching and the Melanoma Microenvironment; Impact on Immunotherapy and Drug Resistance

**DOI:** 10.3390/ijms24021601

**Published:** 2023-01-13

**Authors:** Sultana Mehbuba Hossain, Michael R. Eccles

**Affiliations:** 1 Department of Pathology, Dunedin School of Medicine, University of Otago, Dunedin 9016, New Zealand; 2 Maurice Wilkins Centre for Molecular Biodiscovery, Level 2, 3A Symonds Street, Auckland 1010, New Zealand

**Keywords:** melanoma, phenotypic plasticity, epithelial–mesenchymal transition, heterogeneity, resistance, MITF, immunotherapy

## Abstract

Melanoma, a highly heterogeneous tumor, is comprised of a functionally diverse spectrum of cell phenotypes and subpopulations, including stromal cells in the tumor microenvironment (TME). Melanoma has been shown to dynamically shift between different transcriptional states or phenotypes. This is referred to as phenotype switching in melanoma, and it involves switching between quiescent and proliferative cell cycle states, and dramatic shifts in invasiveness, as well as changes in signaling pathways in the melanoma cells, and immune cell composition in the TME. Melanoma cell plasticity is associated with altered gene expression in immune cells and cancer-associated fibroblasts, as well as changes in extracellular matrix, which drive the metastatic cascade and therapeutic resistance. Therefore, resistance to therapy in melanoma is not only dependent on genetic evolution, but it has also been suggested to be driven by gene expression changes and adaptive phenotypic cell plasticity. This review discusses recent findings in melanoma phenotype switching, immunotherapy resistance, and the balancing of the homeostatic TME between the different melanoma cell subpopulations. We also discuss future perspectives of the biology of neural crest-like state(s) in melanoma.

## 1. Introduction

Malignant melanoma is one of the most aggressive, heterogeneous and treatment-resistant cancers [1], which exhibits among the highest mutation rates when compared to other cancers [2]. Malignant melanoma is highly heterogeneous and is associated with multiple transcriptionally distinct cell phenotypes. These distinct phenotypes are plastic in nature and can switch from one phenotypic state to another to escape both targeted therapy and immunotherapy [3].

Approximately 75% of deaths from skin cancer are due to malignant melanoma. While the 5-year survival rate is over 90% for localized melanoma, this drops to 16% for distant disease, indicating that metastasis is the main reason for poor outcomes [4]. Recent advances in genomic technologies, especially single cell-based sequencing, and the availability of online gene expression datasets, have allowed the characterization of the different subsets of melanoma based on gene signatures and enabled categorization of them as distinct phenotypes.

Melanoma originates from the malignant proliferation of the pigment-producing melanocyte cells, and occurs predominantly in fair-skinned populations, although it can affect darker-skinned populations as well. Cutaneous melanoma predominantly occurs due to excessive ultra-violet irradiation exposure and mutations occurring in the melanocyte cells located in the basal layer of the epidermis in the skin. Melanoma occurs as well on the acral skin on the palms of the hand and soles of the feet to which darker-skinned populations are also susceptible. Additional forms of melanoma occur in the mucosal surfaces, the uveal tract, and leptomeninges [5,6]. The Clark model for melanoma progression depicts a series of histopathological transformations of melanocytes in progressing to malignant melanoma and the subsequent development of invasion and metastasis [7], involving the tightly regulated switching of cellular phenotypes. Melanoma exhibits characteristics in many measurable traits from early to advanced stages, including proliferation, metastatic potential, and therapeutic resistance. The progression from non-invasive to invasive melanoma resembles epithelial–mesenchymal transition (EMT), a well-characterized process of phenotypic change that is associated with metastatic progression in epithelial cancers [4]. Both genetic and epigenetic changes contribute to the transformation of melanocytes into metastatic malignant melanoma cells [5,6]. Much, however, remains unknown about melanoma tumor heterogeneity, and its role in disease progression and treatment response.

Targeted therapy and immune checkpoint inhibitors (ICI) are the major treatment options for metastatic melanoma [8,9]. Once melanoma has metastasized, treating metastatic melanoma is very challenging. Irreversible genetic events, such as mutations, are believed to play a key role in melanoma initiation, potentially leading to targeted therapy resistance. However, oncogenic mutations are not responsible for the entirety of dynamic changes observed at the transcriptional and phenotypic level during melanoma progression. A multitude of distinct stable phenotypic cell states can arise within the same melanoma during metastatic dissemination [10], which are unique in terms of molecular and functional profiles, and these characteristics vary between each cell type. Moreover, this phenotypic diversity can facilitate interconversion between phenotypic states in response to drug challenges, and melanoma can evolve to form new drug-tolerant phenotypes, making it difficult to treat [11]. In this review we will discuss the phenotypic heterogeneity of melanoma, the role of phenotypic plasticity to induce EMT, and drug resistance, in particular ICI resistance. This review aims to provide a concise summary of the genomic and phenotypic heterogeneity in melanoma.

## 2. Understanding Heterogeneity in the Melanoma Tumor Microenvironment

A better understanding of the cellular, molecular, and spatial heterogeneity within tumors, and in the tumor microenvironment (TME) is essential for improving clinical outcomes. Critically, the success of immunotherapy treatment relies on interactions between cytotoxic T lymphocytes within the TME and tumor cells [12,13,14], while the melanoma tumor bed is composed of millions of cancer cells surrounded by other non-cancerous cells, which each have distinct patterns of molecular characteristics.

The TME, which includes the extracellular matrix (ECM), plays a crucial role in tumor metastasis [12,13,14]. Within the TME, a heterogenous mixture of non-melanoma cells comprises cells such as fibroblasts, endothelial cells, and various immune cells, including tumor-infiltrating lymphocytes (TILs). Additionally, the melanoma cells exhibit the heterogeneous expression of tumor human leukocyte antigen class I antigens (HLA-1), as well as different sub-compartments within the melanoma that variably express many factors, such as programmed death 1-ligand 1 (PD-L1) [12,13,14]. Moreover, extensive molecular differences between individual tumor cells, cell clusters or topographically or anatomically separated tumor lesions are normally observed in melanoma. This degree of heterogeneity contributes to the overall biological behavior of a tumor, disease progression and treatment resistance [15,16].

## 3. Melanoma Phenotypic Plasticity, Melanoma Cell Invasion and Metastasis

**Figure 1:**
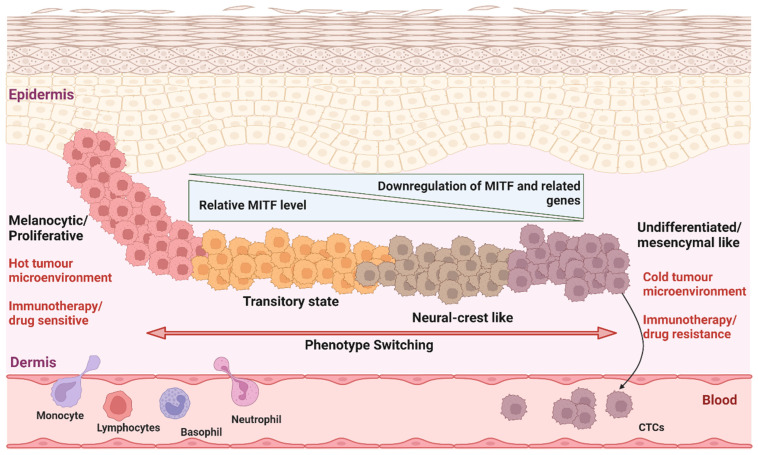
Depiction of phenotype switching during melanoma progression and the development of immunotherapy and drug resistance, based on the expression of *MITF* and *MITF*-related genes in the same tumor bed. Melanoma cells expressing low levels of *MITF* correspond to a slow-cycling and pro-invasive state (similar to “mesenchymal-like”), whereas higher levels of expression of *MITF* correlate with a proliferative and melanocytic state. Undifferentiated melanomas/neural-crest like (on the right side) melanomas lack activated immune cells, while melanocytic melanomas (left side) are composed of immunologically relatively more active immune cells/hot tumor microenvironment, which is also associated with melanocytic melanomas being relatively more responsive to immunotherapy or targeted drugs.

Melanoma is a highly aggressive type of skin cancer [17], exhibiting the ability to switch between different transcriptional states, due to its neuroectodermal origin, with high phenotypic plasticity [18]. Indeed, melanoma’s aggressiveness is at least in part due to its phenotypic plasticity, whereupon early in the tumor evolution, melanoma metastasizes to lymph nodes, distant tissues, and organs, involving the acquisition of functions such as migration and invasion, intravasation, survival in the circulation, extravasation, and colonization at secondary tumor sites [19]. Malignant melanoma cells acquire the capacity to undergo transient and reversible morphological and functional changes by hijacking the embryonic neural crest invasion program, such that metastatic melanoma cells exploit neural crest-related receptor tyrosine kinases to increase plasticity and facilitate invasion, while primary melanocytes may actively suppress these responses under the same microenvironmental conditions [20]. Among the most well-characterized molecular changes that signify a shift in melanoma cell behavior, linked to phenotypic plasticity, and acquisition of migration and invasion, is alterations in the expression level of *MITF*. Very low or absent expression of *MITF* is characteristic of invasive melanoma cells, while high expression of *MITF* characterizes non-invasive melanoma cells [19] (Figure 1).

Thus, melanoma cells can exhibit different phenotypic states based on the heterogeneous expression of *MITF* in the same tumor bed, regardless of whether the cell lines have a mainly invasive (*MITF* negative) or proliferative (*MITF* positive) phenotype in vitro [19] (also see Section 5, below). For instance, Konieczkowski et al. [18] revealed that sensitive melanomas display a high expression of *MITF* and downstream MITF-mediated up-regulation of differentiation markers such as *TYRP1*, *MLANA*, and *PMEL*. The same study also showed that resistant melanomas possess low *MITF* expression, but high levels of inflammatory nuclear factor ‘kappa-light-chain-enhancer’ of activated B-cells (NF-ĸB) signaling and the receptor tyrosine kinase (RTK) *AXL* [18]. That study supported the “phenotype switch” model, as described in the next section—in which adaptive switching between different phenotypes in response to the tumor microenvironment (TME) is crucial for melanoma progression. However, phenotype switching is not obligatory for the invasion and metastatic spread of melanoma cells [21].

Although phenotype switching resembles epithelial–mesenchymal transition (EMT), EMT is not an appropriate term to describe this process in melanoma, as melanocytes are not epithelial cells. Yet, EMT-like processes play a key role during the formation and migration of neural crest cells. Neural crest cells are a multipotent, migratory, transient cell population that migrate through the vertebrate embryo to infiltrate different organs and differentiate in various cell lineages including melanocytes. Melanocytes can be regarded as a product of an embryonic neural crest EMT-like process [21].

## 4. EMT-like Phenotype Switching in Melanoma

As mentioned, melanoma is a highly heterogeneous tumor due to the exacerbated plasticity of tumor cells, which is thought to result from interactions of melanoma cells with the TME, as well as intrinsic tumor cell variability. Tumor regenerative capacity is generally thought to involve rare populations of tumor cells that proliferate slowly, but which retain a high capacity to “seed” new tumor growths. These rare tumor cells possess stem-like properties, and they characteristically conform to the classical cancer stem cell (CSC) hypothesis. However, a seminal study showed that in melanoma, cells with a capacity to seed new tumors were quite common and not rare; therefore, these cells did not fit the classical CSC hypothesis, but rather their frequency would be more compatible with a phenotype switching model [22,23], where reversible epithelial–mesenchymal- and mesenchymal–epithelial-like (EMT-like, and MET-like) transitions are hypothesized to take place. The term “phenotype switching”, which was first introduced by Hoek [19], is becoming increasingly used to describe transitions between phenotypic states [22,24].

EMT and EMT-related phenotypic changes represent an essential biological process during embryonic development, including to provide motility to epithelial cells of the neuroectoderm, neural crest cell migration and melanocyte lineage formation [25,26], which allows melanocytes to emerge from pluripotent neural crest cells [27]. Cellular changes such as those altering cell adhesion, matrix metalloproteinases, and invasive behavior can be induced in in vitro assays, but this does not necessarily overlap with the invasive phenotype defined by gene signatures [3]. EMT and MET (mesenchymal–epithelial transition) processes are mainly controlled by a multitude of cell autonomous and paracrine signals [10]. This evolutionarily conserved process is regulated by embryonic EMT-inducing transcription factors (EMT-TFs), such as SNAIL, TWIST, and zinc finger E-box-binding homeobox (ZEB) protein families and epigenetic regulators [26,28]. However, the EMT process in cancer does not involve a simple shift from a fully epithelial to a fully mesenchymal state, nor is it a binary process. The EMT occurs from distinct intermediate states of cancer cells, which go through a spectrum of multiple states involving reversible transitions, while sustaining cell plasticity [29,30]. However, the acquisition of a fully established invasive phenotype alone is insufficient to cause metastatic disease, because a reversible switch (MET) from an invasive phenotype to a proliferative phenotype is also required for cancer development at the metastatic site. This reversibility of the EMT process depends on multiple factors—(i) proliferative-signature cells being favored over invasive cell types, (ii) proliferative cells being more energetically stable than other types, (iii) microenvironment forces that change the phenotypic signature, and (iv) microenvironment-induced adaptive plasticity [3]. Therefore, while the switch from a proliferative to an invasive phenotype can be induced experimentally, induction of the reverse switch is more difficult.

## 5. MITF and Phenotype Switching in Melanoma

*MITF* is a master regulator of melanocyte development, as well as melanoma cell quiescence, progression, survival, proliferation, and invasion, and even DNA damage repair [31,32]. Expression of *MITF* can be used as a benchmark to distinguish between different melanoma cell states, most often referred to as proliferative and invasive states, or invasive and non-invasive states [19,33,34]. Melanoma cells expressing low levels of *MITF* correspond to a slow-cycling or pro-invasive state (with similarity to “mesenchymal-like”), whereas higher levels of expression of *MITF* correlate with a proliferation state [33,34]. Proliferative melanoma cells are dependent on high glucose levels [35], which leads to hyper-activation of the MAPK pathway, stimulating glycolysis. The glycolysis pathway controls the expression of *MITF* to assist melanoma cell cycle progression [35]. More specifically, during glucose deprivation, melanoma cells decrease *MITF* expression, and become invasive [35]. The same study [35] reported that glucose limitation-dependent regulation of *MITF* levels was not directly linked to a hypoxic response through *HIF1α*. Glucose restriction promotes a transition from a proliferative melanocytic state towards a more aggressive, slow cycling, highly invasive phenotype [36]. This aggressive melanoma phenotype shows low *MITF* and high *AXL* expression, which causes early metastasis through pro-angiogenic factors such as VEGF and interleukin-8 (IL-8) [35].

## 6. The Rheostat Model of MITF

Carreira et al. [37,38] proposed a two-dimensional differentiation trajectory based on *MITF* level—known as the rheostat model. In this model, a lower expression of *MITF* represents reduced melanoma cell proliferation together with increased migration and invasion, and a higher expression of *MITF* denotes the proliferation of melanoma cells. Alternatively, this model can easily be pictured as a bell curve plot where low levels of *MITF* are associated with a G1 arrested or slow-cycling state of melanoma cells, and simultaneously possess high levels of the p27 cyclin-dependent kinase inhibitor, leading to invasiveness. In contrast, a higher expression of *MITF* promotes proliferation and suppression of invasiveness. It is also claimed that a further increase in *MITF* activity can lead to a differentiation associated G1 arrest. This model proposes a pro-proliferative role for *MITF*, driving an invasive to proliferative transition, but also, at very high *MITF* expression levels, an anti-proliferative function in promoting a proliferative to differentiation switch.

Investigating mRNA expression levels, as determined by microarray or RNA-Seq gene expression analysis, several different groups in the late 2000s identified sets of genes in melanoma cell populations exhibiting changes in their gene expression patterns, either positively or negatively in concert with changes in *MITF* levels, and categorized those gene sets as “invasive” and “non-invasive” [33] and “proliferative” and “invasive” [34]. Later in 2018, Tsoi et al. [39] revealed that melanoma cell lines in culture reflect four major clusters, or phenotypic states that can be defined by their gene expression profile—an undifferentiated subtype, a neural crest-like subtype, a transitory subtype, and a melanocytic subtype. This study validated Hoek’s cohort A (melanocytic) and cohort B (transitory), and sub-categorized Hoek’s cohort C into two subtypes distinguished by the expression of the transcription factors SOX10 (neural crest-like, representing cellular invasiveness), and SOX9 (undifferentiated, involving altered cell adhesion and motility). Both undifferentiated and neural crest-like states shared similarities with the invasive state proposed by Hoek (*MITF^low^*, *AXL^high^*) [40]. Clusters possessing low levels of *MITF,* and high levels of *AXL* had elevated *SMAD3* expression, indicating a role for TGF-β signaling. However, in the undifferentiated subtype, significantly lower levels of *ERBB3* and the neural crest marker *NGFR*, together with transcription factor *SOX10*, were observed, exhibiting an upregulation of *SOX9* and *EGFR* genes. In contrast, in the neural-crest like cluster, the neural crest lineage-specifying transcription factors, *SOX10*, *NGFR* and *ERBB3*, were upregulated.

The transitory subtype represents a mixed, transitional phenotype between neural crest and melanocytic subtypes. This state shows intermediate features, expressing *MITF*, but also having several characteristics of neural crest-like cells. This subtype has strong similarities with Hoek’s cohort B [40] and supports the hypothesis that intermediate states might exist in the context of phenotype switching. In the melanocytic subtype, the cells lose a neural crest gene expression signature and gain the expression of pigmentation-associated gene sets. Finally, the transitory and melanocytic subtypes show a higher expression of *MITF* and lower expression of *AXL* [39]. Collectively, these data point toward the expression of *MITF* as a hallmark of specific phenotypic states and are consistent with observations that a sub-population of stochastically generated, slow-cycling, *MITF*-low cells within cultured melanoma cell lines are enriched in cells with an enhanced survival capacity [41].

These findings were extended later by Rambow et al. [42] using single-cell RNA-Seq (scRNA-Seq), which was carried out on ex vivo dissociated melanoma cells obtained from patient-derived xenograft tumors in mice. They noted four distinct co-existing drug-tolerant transcriptional states in response to MAPK inhibitor exposure, which they termed invasive, neural crest stem cell (NCSC), “starved”-like melanoma cells (SMC), and pigmented melanoma cell populations. The invasive cell state exhibited typical characteristics of the undifferentiated state, such as low levels of *MITF* expression and high expression of *AXL*. The NCSC state additionally showed low levels of *MITF* and had a neural crest stem cell-like transcription profile. The SMC state presented an intermediate expression level of *MITF*, but also showed strong down-regulation of the cancer cell metabolic signature with features of nutrient-deprivation, such as enhanced expression of the fatty acid translocase CD36. A fourth state corresponded to cells with a relatively high expression of *MITF*, exhibiting high expression levels of melanocytic differentiation and pigmentation markers, and therefore was defined as pigmented. All four populations were observed in tumor lesions using multiplex immunohistochemistry. Interestingly, the authors tracked the cell subpopulations, including a proliferative subpopulation expressing high *MITF* levels (called “proliferative”) during the development of drug tolerance. Upon drug treatment, the highly proliferative *MITF* cells entered a transitory state (SMC), which preceded a branchpoint, following which the cells entered either a high-*MITF* pigmented state, or a low-MITF NCSC or invasive state. In both drug-naïve patient melanomas and untreated PDX melanomas, NCSC cells were initially relatively rare, but then following the cell state transitions during BRAF/MEK inhibitor treatment, the NCSC population was enriched, although this subpopulation was reduced in numbers again upon drug withdrawal [42]. Understanding the contribution of the different melanoma states to melanoma progression, and how cells can switch from one state to another is a key research area to address in the future.

### 6.1. Phenotype Switching as a Driver of Metastasis

In 2006, Hoek et al. described the existence of two distinct transcriptional and phenotypic states in melanoma [19,40]. Hoek and colleagues [19] first showed that phenotype switching occurs in melanoma following the engrafting of proliferative and invasive cell lines as tumor xenografts in mice. Regardless of the initial phenotype of the cell lines (i.e., proliferative, or invasive), the resulting tumors exhibited both proliferative and invasive cell states, supporting the notion of switching from one state to another [19]. In a later study, Tuncer et al. in 2019 [21] revealed that the modulation of *SMAD7* levels induces phenotype switching during tumor progression. SMAD4 mediates overall canonical TGF-β signaling, being required for melanoma cell proliferation in vivo. In this regard, a TGF-β family member, BMP7, stimulates melanoma cell proliferation, and overrides the antiproliferative and pro-invasive activity of TGF-β and NODAL. In contrast, this invasive character could be triggered through TGF-β-mediated *SMAD7* depletion, which leads to high *AXL* and *ZEB1* expression in cells maintaining high *MITF* expression and proliferative capacity. Consequently, the increase in the number of *MITF^high^AXL^high^* cells in *SMAD7*-depleted melanoma was associated with massive formation of metastases in vivo. This finding demonstrates that proliferation is compatible with increased invasiveness, and the continued expression of *MITF*, revealing TGF-β signaling as a mechanism that underpins melanoma aggressiveness. Another study [43] confirmed the dual role of TGF-β in melanoma, showing that TGF-β normally acts as a tumor suppressor, but during advanced stages of cancer progression, cells are resistant to TGF-β-induced growth arrest and TGF-β becomes a tumor promotor, both in melanoma and epithelial cancers. Additionally, TGFβ can induce the expression of SNAI1, TWIST1 and ZEB family transcription factors, in both a SMAD-dependent and -independent fashion [43].

The heterogeneity present in the melanoma tumor bed has been well-characterized by single-cell RNA sequencing [44]. This has been done for 4645 single cells isolated from 19 melanoma patients. In that study, the authors revealed that malignant cells within the same tumor display transcriptional heterogeneity with respect to cell cycle, spatial context, and drug resistance. Mostly, this analysis categorized melanoma tumors into two distinct transcriptional cell states, such that tumors characterized by high levels of the *MITF* transcription factor were found to also contain cells with low *MITF* and elevated levels of the *AXL* kinase. However, the presence of a small subpopulation of *MITF^high^AXL^high^* cells in every melanoma tumor sample was also noted. Widmer and colleagues have shown that hypoxia can drive invasion without affecting overall proliferation in cultures of melanoma cells [45]. In another study, invasive cell clusters did not alter *MITF* expression, during a process referred to as cooperative invasion [46].

### 6.2. Phenotypic Plasticity as an Enabler of Melanoma Progression and Therapy Resistance

Advances in our understanding of the molecular mechanisms of tumorigenesis have translated into knowledge-based therapies directed against specific oncogenic signaling targets. These therapies often induce dramatic responses in susceptible tumors. Unfortunately, most advanced cancers, including those with robust initial responses, eventually acquire resistance to targeted therapies and relapse (Table 1). Different studies have shown that melanoma develops resistance to therapy through phenotype switching [47,48,49]. For instance, melanoma cells in vitro were able to adopt transcriptionally different cell populations over time when treated using MAPKi. In the initial phase, the cells were mostly melanocytic in nature (*MITF^high^*), but with time, tumor cells began to show characteristics of dedifferentiation, together with the existence of a slow-cycling neural-crest-like state with the expression of NGFR (*NGFR^high^*) [47,48,49]. Crucially, for patients with melanoma undergoing treatment with both a BRAF inhibitor (BRAFi) and a MEK inhibitor (MEKi), the respective *NGFR^high^* and *MITF^high^* transcriptional states in the tumor coexist at the initial drug-response phase, although there is clear interpatient variability regarding the predominant states [42].

Although immune checkpoint inhibitor-based therapies are somewhat more promising than targeted therapies in achieving complete cures, for most patients, intrinsic or acquired resistance remains a key obstacle. Hossain et al. (2022) [50] showed that non-responding melanomas to anti-PD1 therapy are enriched for genes characteristic of undifferentiated and neural crest-like differentiation states, while in contrast, responding melanomas to anti-PD1 therapy exhibit expression signatures mainly characterized by transitory and melanocytic state gene signatures, supporting the notion that phenotype switching behavior characterizes responding versus non-responding melanomas (Figure 1). The same study also showed an association between phenotype switching, immune cell composition in the TME, and the presence of innate resistance to anti-PD1 therapy. The heterogeneity of immune cell composition in the non-responding tumor bed, especially with respect to increased numbers of M2 subtype macrophages, was associated with remodeling of the ECM, suppression of the immune system, and accumulation at the invasive tumor edge, the cancer cell/stromal border, the central tumor mass, and the perivascular areas [51]. Acquired resistance is a direct consequence of pre-existing intratumoral heterogeneity and ongoing diversification during therapy, which enables some tumor cells to survive treatment, and facilitates the development of new, therapy-resistant phenotypes [15].

**Table 1:** Currently available treatment strategies for cutaneous melanoma.

Group	Drug Name	Target	Mechanism of Action	References
ICI therapy	Ipilimumab	CTLA4	Primes anti-tumor immune response by inhibiting the cytotoxic T-lymphocyte-associated protein 4 (CTLA-4) signaling pathway.	[52]
	Pembrolizumab/Nivolumab	PD-1	Blocks the binding of PD-1 with its ligand PD-L1, in turn causing T cell activation and restoration of antitumor activity.	[53,54]
	Atezolizumab	PD-L1		
Targeted therapy	Dabrafenib/Vemurafenib/Encorafenib	BRAF^v600E/K^ mutation	Acts on melanomas that have V600E or V600K mutations in the BRAF protein, as a potent inhibitor of BRAF through ATP competitive binding of the active conformation of BRAF kinase.	[55]
	Trametinib/ Cobimetinib/Binimetinib	MEK	Interferes with abnormal BRAF signals to slow or stop out-of-control cell growth.	[56]
Combination targeted therapy	Dabrafenib + Trametinib	BRAF^V600E/K^ mutant, MEK	Inhibition of the BRAF-MEK pathway, and decreased tumor cell survival in BRAF-mutants.	[57,58,59]
	Vemurafenib + Cobimetinib			
	Encorafenib + Binimetinib			
Combination of ICI and targeted therapy	Pembrolizumab + Dabrafenib + Trametinib	PD1, BRAF^V600E/K^, MEK	Blocks programmed death 1 (PD-1) and enhances the durability of anti-tumor responses by combined inhibition of mutant BRAF^V600^ and MEK signaling pathways.	[60]
	Atezolizumab + Vemurafenib + Cobimetinib	PD-L1, BRAF^V600E/K^, MEK	Combination BRAF plus MEK inhibitors and immune checkpoint inhibitor therapy in BRAF^V600^ mutation-positive advanced or metastatic melanomas.	[61]

### 6.3. Inducers of EMT-like Phenotype Switching in Melanoma

EMT-TFs such as SNAIL, TWIST, and ZEB protein family members in epithelial cancer types directly or indirectly inhibit tumor suppressor protein E-cadherin to induce EMT [25,28]. E-cadherin is a cell-adhesion glycoprotein, which is encoded by the *CDH1* gene. The lower expression of *CDH1* leads to the loss of intercellular junctions, expression of the intermediate filament protein, vimentin, as a major component of the cytoskeleton in mesenchymal cells, and the expression of N-cadherin (encoded by the *CDH2* gene) [62]. The upregulation of vimentin and matrix metalloproteinases can also be caused by tumor necrosis factor-α (TNFα), a proinflammatory cytokine which induces *SNAI1*, *SNAI2*, *TWIST1*, and *ZEB* expression via NF-κB [63]. However, Caramel et al. (2013) [64] showed that the regulation and functions of phenotype switching-associated transcription factors (as opposed to EMT-TFs) are different in malignant melanoma. Unlike EMT, *ZEB1* and *ZEB2* are differentially expressed in alternate phenotypic states [64]. Furthermore, expression of *ZEB1* and *TWIST1* promote malignant changes, whereas *SNAIL2* and *ZEB2* transcription factors are expressed in normal melanocytes and behave as tumor-suppressor proteins by activating the microphthalmia-associated transcription factor (MITF)-dependent melanocyte differentiation program. While down-regulation of *MITF* leads to an invasive phenotype [19], the loss of *ZEB2* in melanocytes leads to dedifferentiation, and in melanoma cells results in increased *ZEB1* expression, repressing E-cadherin, and contributing to progression and metastasis [65]. These studies suggest that *ZEB2* functions as a differentiation factor, through the maintenance of E-Cadherin expression [65]. Another study compared the gene expression patterns between non-metastatic and metastatic melanoma samples and revealed that loss of E-cadherin/gain of N-cadherin is a major determinant of melanoma metastasis [66].

The cytosolic distribution of β-catenin does not play a direct role in melanoma phenotype switching. Instead, the expression of two β-catenin co-factors, *LEF1* and *TCF4*, were found to be phenotype-specific and inversely correlated [67]. Differentiated/proliferative phenotype melanoma cells preferentially express *LEF1* and dedifferentiated/invasive phenotype cells usually express *TCF4*. The same study showed that loss of *LEF1* and gain of *TCF4* expression was associated with melanoma progression, involving changing from a proliferative to an invasive phenotype [67]. The beta-catenin/LEF1 complex is regulated by WNT signaling and leads to activation of *MITF* [68].

The observation that in one phenotypic state melanoma cells proliferate rapidly but invade poorly, while in the other phenotypic state melanoma cells proliferate slowly, but are more invasive, has been confirmed in 3D cultures and xenografts [48,69]. Evidence has been provided that these cells switch back and forth between these two states, which are referred as “proliferative” and “invasive/mesenchymal-like” states, also known as “melanocytic” and “undifferentiated” [70].

Melanocyte cell proliferation and differentiation from neural crest precursors relies heavily on the canonical WNT signaling pathway, through β-catenin as an intermediate molecule, in the regulation of *MITF* [71]. Activation of β-catenin in melanoma primarily occurs via mutation or deregulation, and ultimately it is crucial for bypassing melanocyte senescence, leading to the transformation of melanocytes [72]. In contrast, the invasive or mesenchymal phenotype depends on WNT5A, which is a non-canonical signaling pathway that activates protein kinase C and stimulates the release of intracellular calcium, resulting increased motility and transition [73,74]. WNT5A also downregulates *MITF* expression, eventually promoting a metastatic phenotype [75]. Invasive melanoma cells require remodeling of their cytoskeletal organization for cellular transformation, as well as alteration in their response to the ECM and the surrounding stromal cells, in order to gain access into lymphatic vessels. Tumor cells ultimately migrate to lymph nodes and enter into the systemic circulation [76,77,78]. WNT5A elevates β-catenin degradation which is critical for melanoma invasion and metastatic outgrowth [79], and again is, required for switching from an invasive phenotype to re-enter into proliferative state after distant metastasis [80]. However, the tumor microenvironment also plays an important role in governing the switch to the proliferative state at the metastatic niche.

## 7. Phenotypic Instability and the Tumor Microenvironment

Melanoma interacts with its TME in three different ways—(i) through mutual communication between melanoma cells and the stroma either via cell–cell or cell–matrix contact through cytokines and growth factor secretion, which further leads to remodeling of TME, angiogenesis formation, melanoma growth, metastasis, invasion and migration; (ii) through melanoma cells taking control over their surrounding epidermal tumor microenvironment; and (iii) through the construction of sub-compartments within the tumor for access to oxygen and nutrients [14]. In addition, the TME plays a major role in adaptive phenotypic plasticity by inducing transcriptional changes through signaling pathways. The non-cancer cells in the TME and the metabolic conditions can modify tumor phenotypes and drive them from proliferative to invasive, and vice versa [35,81], which also indicates that melanoma phenotype plasticity is not only responsible for the development of resistance to targeted therapy, but also to immunotherapy [3,42].

Melanoma-associated fibroblasts (CAFs) in the tumor stroma are associated with an increased risk of invasion, metastasis, and poor prognosis of melanoma through the release of a variety of chemokines and cytokines, as well as ECM components and ECM-remodeling enzymes to promote tumor aggressiveness and drug resistance [82]. Moreover, CAFs promote therapeutic resistance and melanoma invasiveness through the secretion of IL-6 and IL-8 cytokines [82]. Tumor-intrinsic signaling pathways, such as WNT/β-catenin signaling, STAT3 signaling, p53 signaling, and RAS/RAF/MAPK and JAK2/STAT1 signaling pathways, have a key role in regulating the immunosuppressive tumor microenvironment and in tumor immune escape [83,84].

β-catenin is frequently highly expressed in melanoma and inhibits immune cell activation to mediate melanoma immune escape [85,86]. Another transcription factor, signal transducer and activator of transcription 3 (STAT3) is aberrantly activated in tumor cells, and it is immunosuppressive in advanced disease stages as a regulator of hypoxia-inducible factors HIF-1α and growth factors to improve cancer development [87,88]. STAT3 is a negative regulator of immune helper T cells, and decreases the expression of proinflammatory factors, including the chemokines CCL5 and CXCL10 to interfere with T cell recruitment [86,89]. Additionally, STAT3 helps tumor cells to escape immune cycle by promoting transforming growth factor-β (TGF-β), VEGF, MDSC and suppressing NK cell function [87]. Besides, STAT3 stimulates the polarization of tumor-associated macrophages (TAMs) towards the M2 phenotype, as well as PD-L1 expression, which also facilitates tumor progression [90].

The PI3K/PTEN/AKT/mTOR pathway interferes with the host immune response and helps in tumor immune escape. Hyperactivation of the PI3K/AKT/mTOR pathway is involved in the upregulation of IL-8 and VEGF [91]. With the activation of PI3K, the expression of PD-L1 increases, which then decreases the function of Treg cells [92]. Increased PD-L1 levels can also lead to the accumulation of TAMs to induce an immunosuppressive microenvironment [84]. PTEN is another tumor suppressor, the inactivation of which is associated with the lack of T cell infiltration as well as low PD-L1 expression in the TME [93]. Furthermore, oncogenic activation of the AKT-mTOR signaling pathway promotes immune escape by driving PD-L1 expression [94].

However, none of these studies considered the extent to which the different cell states switch from one to the other, and whether they transition to ‘pseudo-intermediate’ or stable cell states while overcoming barriers in the process of dissemination. Investigating this may require single-cell multi-omics methods to be used to assess the (epi)genomic and transcriptomic identity to understand the magnitude of cell plasticity and gene regulatory networks that govern each state during dissemination. Spatial mapping of the phenotypic cell states would further improve our understanding of possible niches that (de)differentiated cell states preferentially acquire and how the tumor microenvironment facilitates their switch from one state to another.

## 8. Signaling, Dedifferentiation and Developmental Pathways Involved in Melanoma Phenotype Switching

Melanoma onset and progression involves alterations in signaling pathways, including mitogen-activated protein kinase (MAPK), protein kinase B (AKT) and p53 pathways [95], which affect processes such as cell-cycle regulation, pigmentation, as well as others. In advanced stages, melanomas metastasize to other organs through several key steps such as invasion, intravasation, circulation, extravasation, and colonization at secondary tumor sites [96,97], which are orchestrated by a series of distinct steps [98]. Metabolic reprogramming, such as cellular metabolism, lipid metabolism, amino acid metabolism, nucleotide metabolism, oxidative phosphorylation, and autophagy, are crucial characteristics for the metastatic process [99]. Inflammatory signaling pathways are also a major contributor to melanoma progression, often by creating an environment that is conducive to melanoma-genesis [95]. Inflammatory factors including tumor necrosis factor (TNFα), IFN-γ, interleukins, and related regulatory signaling such as Janus kinase (JAK)-STAT, and NF-κB. The role of inflammatory signaling pathways in melanoma has been reviewed in detail by Guo et al. [95], and here we will only provide a snapshot of the role of signaling pathways in melanoma phenotype switching (Table 2).

Melanoma exhibits many similarities to melanocyte precursors, suggesting that melanoma may use similar transcription factors and regulators as those involved in embryonic signaling pathways to facilitate melanoma progression and tumorigenic functions [100,101]. For instance, *SOX10*, *MITF*, Notch, and WNT-β-catenin, transcriptional factors and signaling pathways are well-characterized for their role during neural crest cell development, and the formation of the melanocytic lineage, and these factors are involved in the malignant characteristics of melanoma cells [68,102].

**Table 2:** Role of signaling pathways in melanoma phenotype switching.

Pathways	Role in Melanoma Phenotype Switching
RAS/RAF/MEK/ERK pathway	Mutations in *BRAF*, *NRAS*, *NF1* and *KIT* occur commonly during cutaneous melanoma carcinogenesis, which cause hyper-activation of the RAS/RAF/MEK/ERK pathway [103]. This pathway is responsible for regulating cancer cell proliferation, survival, and apoptosis, and is mainly controlled by phosphorylation and de-phosphorylation, involving phosphatases, GTP/GDP exchange proteins, adaptor proteins and scaffolding proteins [103,104]. For instance, the *BRAF*^V600E^ mutation leads to elevated activation of MEK and ERK [105], which then facilitate changes in cell adhesion, cell cycle progression, cell migration, cell survival, differentiation, metabolism, proliferation and transcription [106,107,108,109,110]. Likewise, mutations in *NRAS* cause prolongation of the GTP binding, which activates RAF proteins, leading to enhanced signaling via the MAPK pathway.
Nuclear factor κ-B (NF-κB) signaling	Mutations in *NRAS*, for example, use CRAF to activate the MAPK pathway in melanoma [111,112]. Activation of ERK regulates melanoma cell survival, proliferation, resistance to apoptosis and metastasis though phosphorylation and activation of nuclear factor κ-B (NF-κB) transcription factor [113], which further induces Snail, promoting a mesenchymal phenotype in epithelial cells [114] and in melanoma [115].
JAK/STAT pathway	The JAK/STAT pathway interacts with receptor tyrosine kinase (RTK) pathway signaling at multiple levels, leading to mutual activation. Further, the activation of RTK signaling causes activation of EGFR and downregulation of MAPK, which in turn phosphorylates STAT to promote JAK/STAT signaling pathway activation. In addition, RTK and PI3K pathways crosstalk with TGF-β signaling to interact with the JAK/STAT signaling cascades, which are activated upon cytokine stimulation [116].
TGF-β signaling pathway	TGF-β is the most extensively studied inducer of EMT, with established roles in regulating ECM remodeling and in influencing cell phenotype [117,118]. TGF-β signals through SMAD3 to activate *SNAI2*/*SLUG* in a Rho-pathway dependent manner [119]. Enhanced TGF-β signaling is also involved in facilitating resistance to targeted inhibition of many oncogenic signaling pathways [120].
WNT/β-catenin signaling pathway	The WNT/β-catenin signaling pathway is very important in embryonic development and in adult tissue homeostasis, cell migration, hematopoiesis and wound repair. In addition to the WNT/β-catenin canonical pathway, WNT glycoproteins can also activate WNT/Ca^2+^ pathways, involving activation of protein kinase C (PKC), and the WNT/planar polarity pathway, which involves activation of c-Jun N-terminal kinases (JNKs) [121,122]. WNT5A and protein kinase C (PKC) expression is associated with tumor aggressiveness, cell proliferation and invasiveness, and decreased differentiation. WNT5A/PKC also stimulates melanoma cell motility via induction of genes involved in the EMT program of carcinomas, including downregulation of E-cadherin [123].
IFNγ	IFNγ plays an important role in reshaping the melanoma microenvironment, especially in relation to pro-survival and immune evasion effects involved in ultraviolet radiation-induced melanoma-genesis. IFN-γ pathways have a dual role in the anti-tumor immune response—producing an effective anti-tumor immune response by directing anti-proliferative and pro-apoptotic effects on tumor cells, enhancing the expression of MHC and other molecules to increase tumor neoantigen presentation, and by recruiting other immune cells [124]. Also, IFN-γ can cause immune escape by increasing the expression of PD-L1 on the surface of tumor cells [125]. IFN-γ is mainly produced by Th1 cells, and is critical for the immune response, and for sustained M1 macrophage bioactivity to eliminate neoplastic cells [126].

## 9. Balancing Melanoma Heterogeneity

Tumor cells receive extracellular signals, as well as microenvironmental stresses, which cause DNA damage responses, unfolded protein responses, and mitochondrial stress signaling [127]. Ultimately, these factors lead to tumor heterogeneity, and they affect the histological and vascular architecture [128]. The TME directs melanoma cells into a range of phenotypic states that may coexist in varying proportions—some cells may be differentiated and reflect the specialized function of the cell of origin, whereas a proportion of cells are actively cycling, and thus fuel tumor growth, and a third class of cells will be invasive, some of which may have the potential to seed new metastases. Finally, dormant cells may lie quiescent for many years before their reactivation, when they may initiate a new tumor (i.e., metastatic lesion) or give rise to relapse after an apparently successful therapy [129,130]. Simultaneously, the immune component in the TME can also adapt the extrinsic stimuli, based on oxygen tension, glucose availability, or oxidation pathways [131], leading to reprogramming of the TME [132]. Therefore, it is important to determine how the different melanoma phenotypic states, and especially the intermediate/transitory state, are initiated and maintained, how they influence tumor progression, and whether they exhibit any unique therapeutic vulnerabilities.

Wouters et al. [133] designed a model to potentially distinguish between the melanocytic and intermediate cell states and defined the gene regulatory networks that maintain phenotypic diversity in melanoma. Single-cell mRNA sequencing was used to confirm three distinct melanoma cell states in patient-derived cultures—melanocytic lineage behavior, or neural crest-like properties were stratified by expression of *SOX10*. De-differentiated or mesenchymal cell states were categorized by differential expression of the *SOX9* expression marker, and all other melanocytic cell states were determined by *TFAP2A* expression. Using principal component analysis (PCA), the authors then revealed that PC1 confirmed a clear melanocytic–mesenchymal axis, whereas PC2 revealed that gene expression was influenced by an immune-response-like program, and suggested a distinct intermediate cell state that was characterized by the co-expression of melanocytic (*SOX10*, *MITF*, and *TYR*), mesenchymal (*FN1* and *S100A16*), immune-related (*IFITM3* and *HLA*-B) and neural crest stem cell-related genes (*NES* and *MIA*). The authors noted three distinct cell states in melanoma that differ in their functional abilities, migratory capacity, as well as gene expression. In the absence of *SOX10*, melanocytic cultures experienced marked cell death, demonstrating reliance on this melanocyte-lineage transcription factor for survival, whereas cultures with an intermediate gene expression were less sensitive to *SOX10* depletion. It remains important to determine what external signals guide phenotypic plasticity away from the mesenchymal states, towards an intermediate or melanocytic cell state, which may allow conversion of aggressive melanomas towards less aggressive, or more drug-responsive cell states.

## 10. Communication between Melanoma Cells in Different Phenotypic States

**Figure 2:**
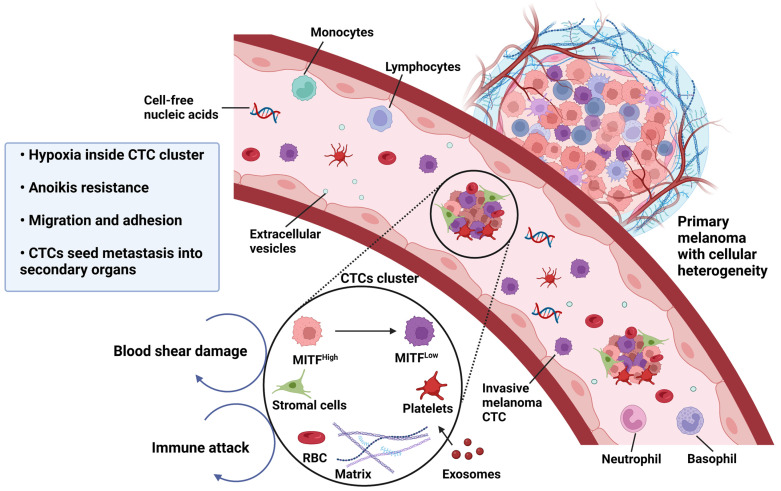
CTC clusters in the bloodstream are heterogeneous, and comprise cells with an *MITF^high^* proliferative phenotype, an *MITF^low^* invasive phenotype, pericytes, immune cells, platelets, and CAFs. The crosstalk between proliferative and invasive melanoma cell types in the CTC cluster facilitates seeding of CTCs and increased metastasis. Melanoma cells within the bloodstream are subject to severe stresses, such as loss of cell anchorage (anoikis), fluid shear stress, oxidative stress, and immune attack. The *MITF^low^* cells participate with *MITF^high^* cells to avoid anoikis, and the aggregation of CTCs in the bloodstream promotes avoidance of sheer forces in blood. Hypoxic conditions in the CTC clusters promotes invasion and endows circulating melanoma cells with a reactive oxygen species (ROS)-resistant phenotype that enhances CTC survival.

The phenotype switching model is not only limited to cells in the tumor bed undergoing metastasis. Phenotypic heterogeneity has also been observed in circulating melanoma cells [44,134]. Notably, in circulating tumor cells (CTCs), heterogeneity was confirmed through analysis of *MITF* expression levels within circulating melanoma cell clusters [134] (Figure 2). Melanoma cells in the circulation survive detachment (anoikis). Moreover, circulating melanoma cells with the *MITF^low^* invasive phenotype expressing the AP-1 transcription factor subunit *FOSL1* are resistant to anoikis [135]. It was observed that *MITF^high^* cells, which are otherwise prone to anoikis, also appear to receive a survival benefit because of their attachment to *MITF^low^* cells within circulating clusters. These findings suggest that melanoma cells harboring different phenotypes can communicate with each other to maintain a homeostatic balance within the same circulating melanoma cell cluster. Another study has shown that in vivo melanoma cells cooperate and communicate between invasive and proliferative phenotypes to increase invasion efficiency [46].

Moreover, both genetic and epigenetic diversity within a population provide growth and survival advantage to some cells when exposed to specific stress signals [136], despite subpopulations of cancer cells within a given tumor frequently sharing the same driver mutations [137]. Taken together, these findings suggest that the metastatic process is a collaborative act, resulting from different phenotypic populations of melanoma cells.

## 11. Conclusions

Melanoma heterogeneity results from diverse cancer cell evolution during the disease course. Despite intensive research, many aspects regarding melanoma cellular heterogeneity remain unclear. The exact relationship between phenotypically switched melanoma cells, and epigenomic characterization of melanoma subpopulations within patients’ tumors still needs to be clarified. Further, the specific role of communication between different subpopulations within the tumor and the various biological components within the tumor microenvironment (ECM and immune cells) remain incompletely characterized. As progressively more subpopulations of tumor and stromal cells develop in association with melanoma, the more challenging successful treatment becomes, such that in advanced melanoma, treatment frequently follows a pattern involving tumor growth, followed by metastasis, reduced efficacy of therapeutic treatment and, finally, resistance to treatment. Treatment strategies designed for the effective targeting of tumor heterogeneity in melanoma could lead to better therapeutic options and better outcomes for patients.

## Data Availability

No new data were generated in this work.
